# Sex- and the age-specific survival impacts of multiple body composition parameters in patients with gastroesophageal cancers

**DOI:** 10.1007/s00595-026-03458-y

**Published:** 2026-08-07

**Authors:** Kotaro Sugawara, Shinichiro Shiomi, Wataru Gonoi, Satoru Taguchi, Shouhei Hanaoka, Koichi Yagi, Osamu Abe, Haruki Kume, Yoshifumi Baba

**Affiliations:** 1 Department of Gastrointestinal Surgery, Graduate School of Medicine, The University of Tokyo, 7-3-1 Hongo, Bunkyo-ku, Tokyo, 113- 8655 Japan; 2 Department of Radiology, Graduate School of Medicine, The University of Tokyo, Tokyo, Japan; 3 Department of Urology, Graduate School of Medicine, The University of Tokyo, Tokyo, Japan

**Keywords:** Body composition, Low muscle mass, Skeletal muscle index, Subcutaneous adiposity, Visceral adiposity

## Abstract

**Purpose:**

This study aimed to evaluate the sex- and age-specific survival impacts of body composition (BC) parameters in patients with surgically treated gastroesophageal cancer.

**Methods:**

A total of 1382 patients were retrospectively analyzed. The skeletal muscle index (SMI), skeletal muscle radiodensity (SMD), and visceral/subcutaneous adipose tissue index (VATI/SATI) were determined. The survival impacts of the BC parameters were investigated according to age (≥ 75 or < 75 years) and sex. Multivariate Cox hazard models were used to identify independent predictors of a poor overall survival (OS) and cancer-specific survival (CSS).

**Results:**

The median age was 67 years, and 1028 (74.4%) patients were male. A low SMI (*P* < 0.001), a low SATI (*P* = 0.048), and a low SMD (*P* = 0.01) were independent predictors of poor OS in younger patients; however, only a low SMI (*P* < 0.001) had an independent impact on OS in the older group. In male patients, all four BC measures significantly demarcated OS curves (all *P* < 0.01), and a low SMI had an independent impact on CSS (*P* = 0.03). In contrast, in female patients, only SMI and SATI reliably discriminated OS curves (both *P* < 0.001), and a low SATI was an independent predictor of a poor CSS (*P* = 0.008).

**Conclusions:**

The prognostic implications of the BC parameters may vary by age and sex in patients with gastroesophageal cancer.

**Supplementary Information:**

The online version contains supplementary material available at https://doi.org/10.1007/s00595-026-03458-y.

## Introduction

Gastroesophageal cancers (GECs), including gastric and esophageal cancers and cancers of the gastroesophageal junction, are among the most prevalent gastrointestinal malignancies globally [1]. Body composition (BC) evaluation is crucial for optimizing the outcomes of patients with solid tumors, including gastrointestinal malignancies [2].

BC assessments using readily available computed tomography (CT) images can distinguish skeletal muscle, visceral, and subcutaneous adipose tissue, thereby serving to identify hidden BC abnormalities, which may be present even within the normal limits of traditional anthropometric measurements [3]. Numerous studies have suggested that a low muscle mass (myopenia) [4, 5], low muscle quality (myosteatosis) [6], and adipose tissue distribution abnormalities [7], are closely associated with poor clinical outcomes in various diseases, including gastrointestinal malignancies.

Notably, the impact of the BC status reportedly differs according to the physiological status of the patient. Recent studies have shown that the impacts of a low body mass index and low muscle mass differ markedly between older and younger patients with malignancies [8, 9]. Furthermore, BC parameters, especially those related to adiposity, reportedly exert different clinical impacts according to sex [10, 11]. These past investigations prompted us to hypothesize that the survival significance of BC parameters varies according to age and sex in patients with GEC.

The age- and sex-dependent effects of BC parameters have not yet been investigated in patients with GEC. We herein focused on the survival and oncological impacts of muscle mass, muscle quantity, and subcutaneous/visceral adipose tissue volume in a large cohort of patients who underwent surgery for GECs.

## Patients and methods

### Patients

The patient selection process has been described in our previous publication [5]. Briefly, 1382 patients who underwent radical surgery for gastric cancer (GC) (*n* = 917) or esophageal cancer (EC) (*n* = 465) at The University of Tokyo Hospital between January 2006 and December 2018 were included in the analysis after exclusion of patients lacking a usable CT image obtained within 3 months before surgery or sufficient clinical information. At the time of the final follow-up (December 2022), the median follow-up period was 61 months for survivors. This cross-departmental, single-center, retrospective study was approved by the Institutional Review Board of the Graduate School of Medicine and Faculty of Medicine, The University of Tokyo (approval number: 2022046NI). We also obtained permission to reuse previously published material from Elsevier (License number: 5683921215082) [5].

### Body composition analysis

A single axial image at the L3 vertebrae, including both transverse processes, was used to estimate the skeletal muscle area (SMA, cm^2^) and visceral/subcutaneous adipose tissue area (VATA/SATA, cm^2^), as previously described [6]. Two certified radiologists cooperatively analyzed and checked the CT images using a platform for computer-aided diagnosis research, CIRCUS version 1.6.0 (http://circus-project.net), powered by our own deep-learning-based semiautomatic segmentation program, as previously described [6]. Skeletal muscle, visceral adipose tissue, and subcutaneous adipose tissue were identified and quantified based on Hounsfield unit (HU) thresholds (–29 to + 150, -150 to -50, and − 190 to -30, respectively) [4, 12].

The cross-sectional areas were adjusted for height squared to calculate the L3 index [SMI, visceral adipose tissue index (VATI), and subcutaneous adipose tissue index (SATI) in cm^2^/m^2^]]. Skeletal muscle density (SMD) was assessed by estimating the mean HU value of the SMA.

### Cut-off values for BC parameters

We recently showed that the Asian-specific cutoff value for SMI yielded superior predictive ability for mortality in an Asian patient cohort [5]. Accordingly, in the present study, the cut-offs set for SMI were sex-specific values, as previously described (36.2 cm^2^/m^2^ for men and 29.6 cm^2^/m^2^ for women) [5, 13].

A receiver operating characteristic (ROC) curve analysis applying Youden’s index (the maximum points of “sensitivity + specificity-1”) for OS was employed to identify the optimal thresholds for VATI, SATI, and SMD, since Asian-specific cut-offs for the three parameters remain to be determined [14]. The thresholds for VATI and SATI were determined as sex-specific values. Considering that SMD is independent of the patient’s body constitution, SMD cut-offs were non-sex-specific.

### Surgical treatment

Our standard surgical treatments and perioperative management protocols for patients with EC and GC have been described in detail in previous publications [8, 9, 15, 16]. For GC, we determined the extent of gastric resection based on the Japanese GC treatment guidelines [17]. Adjuvant S-1 monotherapy has been employed since 2007 for stage II/III disease, with the exception of patients with pT3N0 and pT1 disease [17]. For EC, our standard procedures consisted of subtotal esophagectomy with mediastinal lymphadenectomy via right thoracotomy or a non-transthoracic, transmediastinal approach [15, 18]. We usually employ 3-field lymphadenectomy for upper and middle thoracic EC, while a 2-field lymphadenectomy has generally been selected for lower thoracic and abdominal EC.

### Exposures

The primary and secondary outcomes of this study were OS and cancer-specific survival (CSS), respectively. OS was defined as the period from surgery until death due to any cause. CSS was defined as the period from the date of surgery until death due to primary cancer. We also assessed patient characteristics, including age (≥ 75 vs. < 75 years), sex (male vs. female), cancer type (EC vs. GC), and tumor stage.

### Statistical analysis

Categorical variables were compared using Fisher’s exact test or the χ2 test as appropriate. Continuous variables were compared using Student’s *t-*test. Survival curves were constructed using the Kaplan-Meier method, and the log-rank test was applied to determine statistical significance. Cox proportional hazards regression models were used for the univariate and multivariate analyses of OS and CSS. Clinically meaningful factors with *P* < 0.1 in a Cox proportional hazard model identified by a univariable analysis were regarded as potential risk factors and were further analyzed by applying a multivariable Cox model. ROC curves were generated using 5- year survival as the endpoint. The area under the curve estimation was used to assess the predictive ability of each index. Statistical analyses were performed using the JMP Student Edition (SAS Institute, Cary, NC, USA).

## Results

### Patient characteristics according to age and sex

The clinicopathological characteristics of the 1382 patients are summarized in Table 1. Our series included 917 (66.4%) GC and 465 (33.6%) EC patients. The median age was 67 years, and 1028 (74.4%) subjects in our cohort were male. The older group had a significantly higher proportion of GC patients than the younger group (*P* < 0.001). Older patients had significantly lower SMI and SMD than younger patients (*P* < 0.001) and significantly higher VATI than younger patients (*P* = 0.01). All BC parameters differed markedly between the sexes (all *P* < 0.001). The pStage did not differ significantly according to age and sex. Overall, the BC parameters of patients with GEC showed marked differences according to age and sex.

**Table 1:** Characteristics of 1382 patients according to age and sex

Variables	Overall (*n*=1382)	Age category			Sex		
		Younger (<75) (*n*=1074)	Older (≥75) (*n*=308)	*p* value	Male (*n*=1028)	Female (*n*=354)	*p* value
Cancer type				<0.001			<0.001
Gastric	917 (66.4)	685 (63.8)	232 (75.3)		633 (61.6)	284 (80.2)	
Esophageal	465 (33.6)	389 (36.2)	76 (24.7)		395 (38.4)	70 (19.8)	0.54
Age, y Median (range)	67 (25-93)			0.07	67 (25-93)	68 (34-92)	
Sex							
male	1028 (74.4)	811 (75.5)	217 (70.5)				
female	354 (25.6)	263 (24.5)	91 (29.5)				
Body composition				0.09			<0.001
BMI, kg/m^2^ Median (range)		22 (13.5-36.3)	21.8 (12.8-29.6)	<0.001	22.3 (13.5-36.3)	20.9 (12.8-30.8)	<0.001
SMI, cm^2^/m^2^ Median (range)		40.37 (20.29-65.14)	37.97 (19.32-65.55)	0.01	41.65 (19.32-65.14)	33.48 (19.56-51.11)	<0.001
VATI, cm^2^/m^2^ Median (range)		27.61 (0.09-126.80)	32.12 (0.29-133.27)	0.29	32.68 (0.18-133.27)	16.53 (0.09-84.99)	<0.001
SATI, cm^2^/m^2^ Median (range)		30.69 (0.07-156.99)	32.19 (0.04-113.41)	<0.001	29.06 (0.07-112.08)	39.56 (0.04-156.99)	<0.001
SMD, HU Median (range)		45.58 (9.69-68.34)	39.35 (10.91-70.33)	0.26	44.91 (13.59-68.34)	42.06 (9.69-70.33)	0.06
pStage							
0-I	669 (48.4)	525 (48.9)	144 (46.8)		488 (47.5)	181 (51.1)	
II	315 (22.8)	232 (21.6)	83 (26.9)		225 (21.9)	90 (25.4)	
III	332 (24.0)	264 (24.6)	68 (22.1)		262 (25.5)	70 (19.8)	
IV	66 (4.8)	53 (4.9)	13 (4.2)		53 (5.2)	13 (3.7)	

Abbreviations: BMI, body mass index; SMI, skeletal muscle index; VATI, visceral adipose tissue index; SATI, subcutaneous adipose tissue index; SMD, skeletal muscle density

### Cut-off values for BC parameters

The thresholds and predictive capabilities of the four BC parameters are presented in Supplementary Table 1. Our cohort was dichotomized by each BC parameter according to age and sex (Supplementary Table 2). The older group had a significantly higher proportion of patients with low SMI and low SMD than the younger group (both *P* < 0.001). A significantly higher proportion of female patients had a low VATI and a low SMD compared with male patients (both *P* < 0.001).

### Survival outcomes according to BC and age

**Fig. 1:**
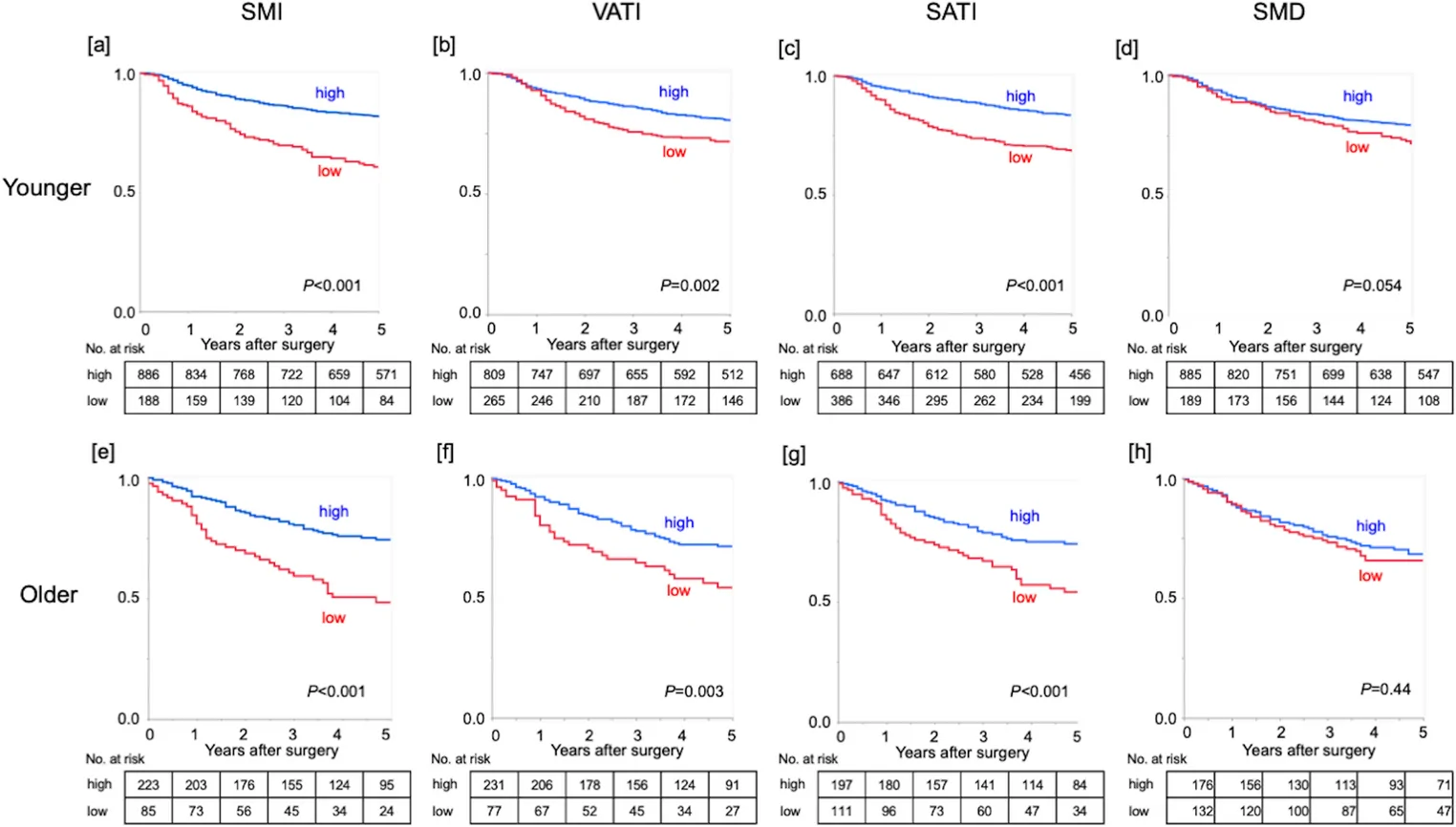
OS curves according to SMI, VATI, SATI and SMD in younger and older patients. OS curves were significantly demarcated by **(a**,** e)** SMI, **(b**,** f)** VATI, **(c**,** g)** and SATI in both younger and older patients (all *p* < 0.01). **(d**,** h)** SMD did not significantly stratify the OS curves

OS curves were significantly demarcated by SMI, VATI, and SATI in both younger and older patients (all *P* < 0.01, Fig. 1). The impact of BC on OS did not differ between patients with GC and EC (Supplementary Fig. 1). The multivariable Cox proportional hazards model revealed that a low SMI (HR 1.71, *P* < 0.001), low SATI (HR 1.39, *P* = 0.048), and low SMD (HR 1.70, *P* = 0.01), in addition to an advanced pStage, were independently associated with a poor OS in younger patients (Table 2). In contrast, only a low SMI was an independent predictor of a poor OS in the older group (Table 2).

**Table 2:** Cox hazards model for overall survival (according to age)

Variables	Univariable analysis			Multivariable analysis		
	HR	95% CI	*P* value	HR	95% CI	*P* value
**Age <75**						
male (vs. female)	1.34	0.99-1.83	0.06	1.19	0.85-1.68	0.31
EC (vs. GC)	2.79	2.17-3.58	<0.001	1.96	1.51-2.55	<0.001
low SMI	2.31	1.75-3.04	<0.001	1.71	1.27-2.29	<0.001
low VATI	1.53	1.17-2.00	0.001	1.11	0.77-1.59	0.57
low SATI	2.04	1.59-2.62	<0.001	1.39	1.00-1.92	0.048
low SMD	1.34	0.99-1.81	0.06	1.7	1.23-2.35	0.001
pStage						
Stage 0-I	Ref			Ref		
Stage II	1.96	1.32-2.90	<0.001	1.67	1.12-2.48	0.01
Stage III-IV	6.21	4.55-8.46	<0.001	5.02	3.66-6.89	<0.001
**Age ≥75**						
male (vs. female)	1.89	1.14-3.12	0.01	1.56	0.89-2.72	0.11
EC (vs. GC)	2.39	1.58-3.64	<0.001	1.67	1.08-2.59	0.02
low SMI	2.46	1.64-3.69	<0.001	2.18	1.39-3.40	<0.001
low VATI	1.88	1.24-2.86	0.003	1.33	0.80-2.18	0.27
low SATI	2.48	1.64-3.76	<0.001	1.49	0.89-2.49	0.13
low SMD	1.17	0.78-1.74	0.45			
pStage						
Stage 0-I	Ref			Ref		
Stage II	1.56	0.88-2.75	0.13	1.41	0.79-2.49	0.24
Stage III-IV	5.02	3.10-8.12	<0.001	4.53	2.76-7.44	<0.001

Abbreviations: EC, esophageal cancer; GC, gastric cancer; SMI, skeletal muscle index; VATI, visceral adipose tissue index; SATI, subcutaneous adipose tissue index; SMD, skeletal muscle density

In younger patients, survival curves were also well-demarcated by SMI, VATI, and SATI when death from primary cancer was taken as the event of interest (all *P* < 0.001, Supplementary Fig. 2a-c). In contrast, only VATI had a significant impact on CSS in older patients (*P* = 0.04, Supplementary Fig. 2f).

In the multivariable analysis incorporating interaction terms between SMI, VATI, SATI, and SMD and age, no interaction terms reached statistical significance (*P* for interaction = 0.17, 0.34, 0.87, and 0.89, respectively), indicating that the prognostic effect of BC did not differ significantly according to age.

### OS outcomes according to BC and sex

**Fig. 2:**
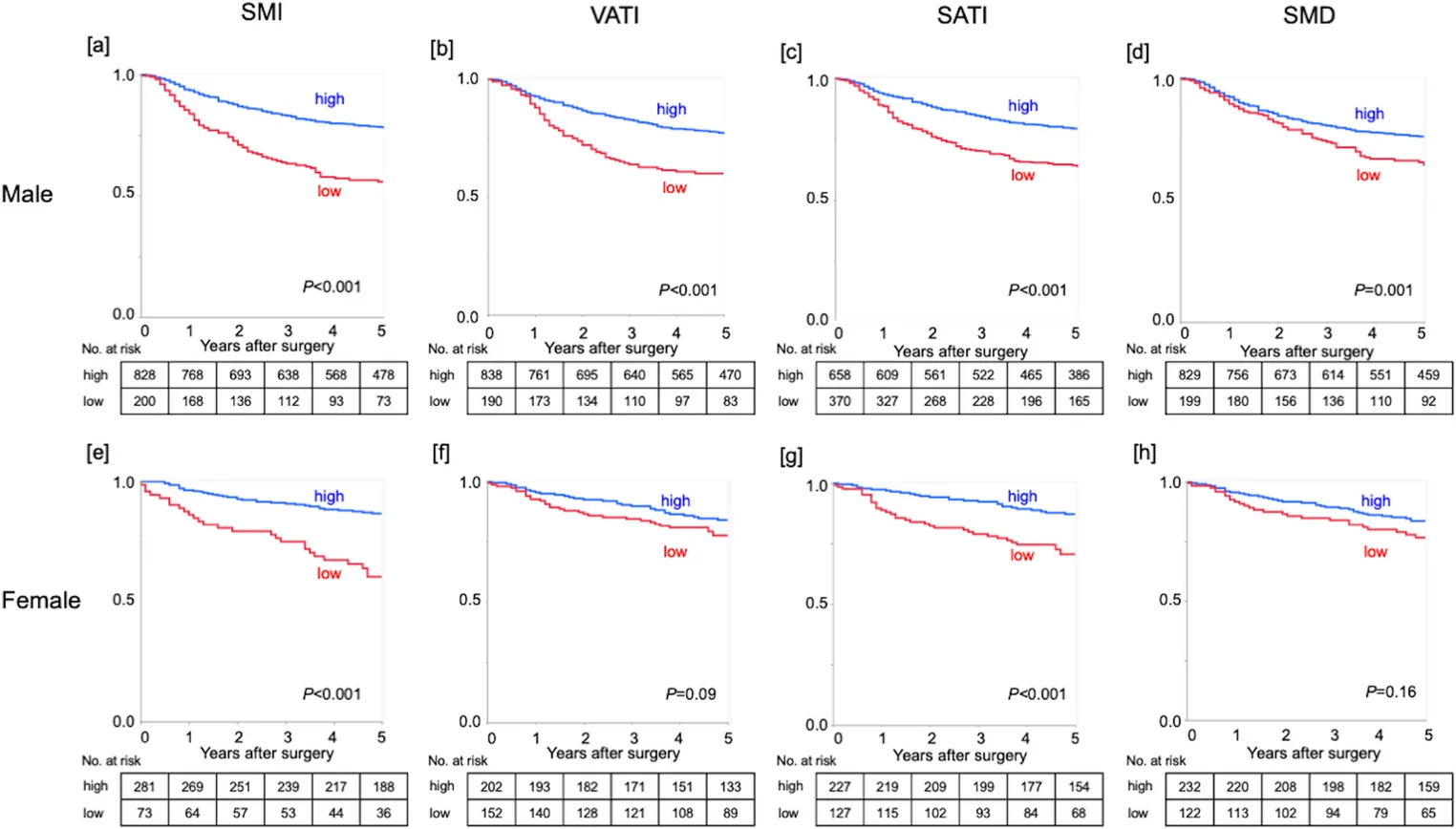
OS curves according to the BC parameters in male and female patients. **(a-d)** OS curves were significantly demarcated by all 4 BC parameters in male patients (all *p* < 0.01). In female patients, **(e)** SMI and **(g)** SATI significantly stratified OS curves (both *p* < 0.01)

Subsequently, we evaluated the impact of the BC parameters on survival by subdividing our cohort into male and female patients. In male patients, all BC parameters significantly demarcated OS curves (all *P* < 0.01, Fig. 2a-d). In females, the OS curves were clearly distinguished only by SMI and SATI (both *P* < 0.001, Fig. 2e, f). In male patients, a low SMI (HR 1.85, *P* < 0.001) and a low SMD (HR 1.74, *P* < 0.001) were independently associated with a poor OS (Table 3), whereas in female patients, a low SMI (HR 1.98, *P* = 0.008) and a low SATI (HR 1.88, *P* = 0.01) were independently associated with a poor OS (Table 3).

**Table 3:** Cox hazards model for overall survival (according to sex)

Variables	Univariable analysis			Multivariable analysis		
	HR	95% CI	*P* value	HR	95% CI	*P* value
**Male**						
Age ≥75	1.61	1.22-2.13	<0.001	1.76	1.31-2.37	<0.001
EC (vs. GC)	2.4	1.89-3.05	<0.001	1.92	1.50-2.46	<0.001
low SMI	2.29	1.77-2.96	<0.001	1.85	1.41-2.44	<0.001
low VATI	1.97	1.51-2.57	<0.001	1.37	0.97-1.93	0.08
low SATI	1.87	1.48-2.38	<0.001	1.21	0.89-1.65	0.22
low SMD	1.55	1.18-2.03	0.002	1.74	1.29-2.32	<0.001
pStage						
Stage 0-I	Ref			Ref		
Stage II	1.79	1.25-2.58	0.002	1.5	1.04-2.16	0.03
Stage III-IV	5.04	3.78-6.71	<0.001	4.5	3.35-6.04	<0.001
**Female**						
Age ≥75	1.37	0.79-2.34	0.25			
EC (vs. GC)	2.47	1.51-4.05	<0.001	1.57	0.95-2.61	0.08
low SMI	3.23	2.00-5.19	<0.001	1.98	1.20-3.27	0.008
low VATI	1.49	0.93-2.39	0.09			
low SATI	2.41	1.50-3.85	<0.001	1.88	1.15-3.06	0.01
low SMD	1.41	0.87-2.27	0.16			
pStage						
Stage 0-I	Ref			Ref		
Stage II	2.33	1.13-4.83	0.02	2.06	0.99-4.29	0.052
Stage III-IV	9.04	4.92-16.63	<0.001	7.31	3.93-13.59	<0.001

Abbreviations: EC, esophageal cancer; GC, gastric cancer; SMI, skeletal muscle index; VATI, visceral adipose tissue index; SATI, subcutaneous adipose tissue index; SMD, skeletal muscle density

A low SATI was not significantly associated with the OS in older women or older men (*P* = 0.07 and 0.11, respectively). In contrast, a low SMI was significantly associated with a worse OS in older men and women (*P* < 0.001 and 0.01, respectively).

In the multivariable analysis, including interaction terms between sex and each body composition (BC) parameter (SMI, VATI, SATI, and SMD), none of the interaction terms were statistically significant (P for interaction = 0.77, 0.39, 0.10, and 0.37, respectively), suggesting that the prognostic impact of BC was consistent between men and women.

### CSS outcomes according to BC and sex

**Fig. 3:**
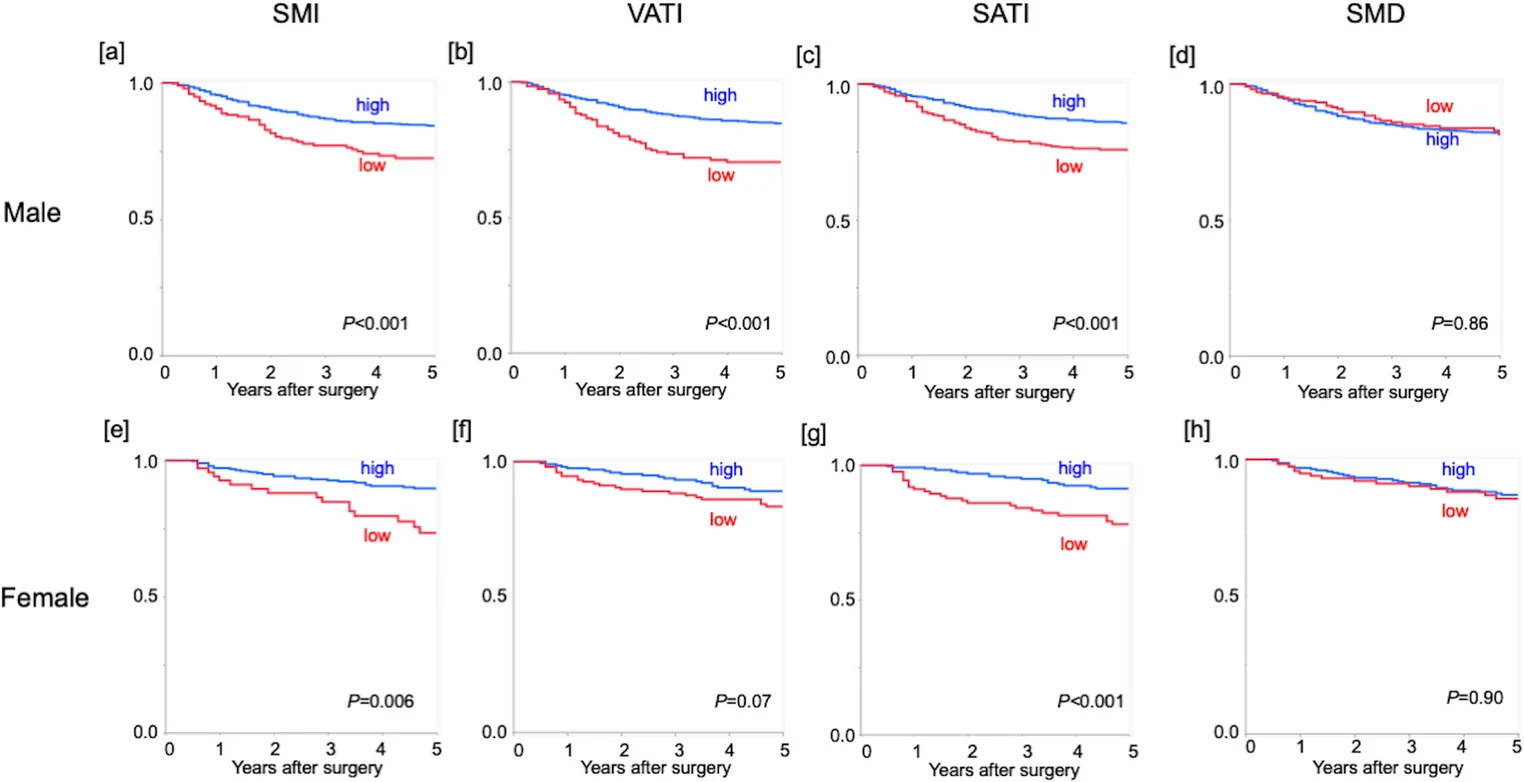
CSS curves according to BC measures in male and female patients. CSS curves were significantly demarcated by **(a)** SMI, **(b)** VATI, and **(c)** SATI in younger patients (all *p* < 0.01). In female patients, **(e)** SMI and **(g)** SATI significantly stratified the CSS curves (both *p* < 0.01)

CSS curves were also well demarcated according to SMI and SATI in both sexes (all *P* < 0.01, Fig. 3). VATI significantly discriminated CSS curves only in male patients (*P* < 0.001, Fig. 3b).

Notably, a low SMI had an independent impact on CSS (HR 1.55, *P* = 0.03) in male patients (Table 4). In contrast, in female patients, a low SATI was an independent predictor of a poor CSS (HR 2.22, *P* = 0.008).

**Table 4:** Cox hazards model for CSS (according to sex)

Variables	Univariable analysis			Multivariable analysis		
	HR	95% CI	*P* value	HR	95% CI	*P* value
**Male**						
Age ≥75	1.14	0.78-1.68	0.5			
EC (vs. GC)	3.33	2.44-4.54	<0.001	2.19	1.60-3.01	<0.001
low SMI	1.88	1.34-2.63	<0.001	1.55	1.08-2.24	0.02
low VATI	2.06	1.48-2.86	<0.001	1.45	0.93-2.25	0.09
low SATI	1.78	1.32-2.39	<0.001	0.95	0.64-1.42	0.81
low SMD	0.97	0.66-1.43	0.86			
pStage						
Stage 0-I	Ref			Ref		
Stage II	4.26	2.36-7.70	<0.001	3.55	1.96-6.43	<0.001
Stage III-IV	15.87	9.55-26.37	<0.001	12.93	7.74-21.61	<0.001
**Female**						
Age ≥75	0.46	0.18-1.16	0.09			
EC (vs. GC)	1.79	0.95-3.38	0.07			
low SMI	2.28	1.25-4.16	0.007	1.3	0.70-2.42	0.4
low VATI	1.67	0.95-2.95	0.08			
low SATI	2.62	1.48-4.63	<0.001	2.22	1.24-3.98	0.008
low SMD	0.96	0.52-1.74	0.9			
pStage						
Stage 0-I	Ref			Ref		
Stage II	3.92	1.31-11.69	0.01	3.69	1.24-11.04	0.02
Stage III-IV	21.35	8.33-54.69	<0.001	19.7	7.66-50.69	<0.001

Abbreviations: EC, esophageal cancer; GC, gastric cancer; SMI, skeletal muscle index; VATI, visceral adipose tissue index; SATI, subcutaneous adipose tissue index; SMD, skeletal muscle density

### Survival outcomes according to BC and cancer type

Given the marked differences in tumor biology, surgical procedures, and perioperative treatment strategies between GC and EC, we further evaluated the survival impact of the BC parameters by subdividing our cohort into GC and EC patients. In the multivariable analysis of OS, low SMI (HR 1.65, *P* = 0.004) and low SMD (HR 1.71, *P* = 0.004) were independently associated with a poor OS in patients with EC (Supplementary Table 3), whereas a low SMI was the only independent predictor of a poor OS (HR 2.28, *P* < 0.001) among the four BC parameters in patients with GC (Supplementary Table 3). In the multivariable analysis of CSS, none of the four BC parameters were independent predictors of a poor CSS, regardless of cancer type (Supplementary Table 4).

## Discussion

The present study demonstrated that the prognostic and oncological implications of the BC parameters vary substantially with age and sex. In younger patients, a broad spectrum of BC measures, including SMI, SMD, and SATI, showed significant survival associations, whereas in older patients, only muscle mass (SMI) retained independent prognostic value. Moreover, we observed clear sex-specific patterns: a low SMI independently predicted poor CSS in men, whereas a low SATI uniquely predicted a poor CSS in women. These findings highlight the need to consider age- and sex-dependent biological features when managing patients with GEC.

In recent years, the clinical relevance of BC has gained attention in the field of surgical oncology. Numerous studies have highlighted the survival impact of weight-based metrics, muscle-associated disorders, and adipose tissue distribution [7–9, 12]. BC abnormalities are reportedly associated with oncological outcomes and responsiveness to immune checkpoint inhibitors [19]. These observations underscore the clinical utility of BC evaluation in patients with malignancies.

Age-related cellular and behavioral changes reportedly induce anabolic resistance, resulting in body fat redistribution and reduced muscle mass [20]. In our study, older patients had significantly lower SMI and SMD and significantly higher VATI than younger patients. These observations, taken together with those of previous studies that showed age-related impacts of BC abnormalities [8], suggest that the clinical relevance of various BC measures differs according to age.

In our study, a low SMI had a robust impact on survival, regardless of age. In contrast, SMD and SATI had a significant impact on OS only in younger patients. These observations suggest that the survival impact of adipose tissue volume and muscle quality decreases with age. Several factors may explain these differences in the results. In older patients, anabolic resistance, mitochondrial dysfunction, and chronic low-grade inflammation collectively blunt the prognostic impact of subtle BC abnormalities [21, 22]. Most older patients already have sarcopenia or a decline in muscle quality, which may diminish the relative influence of parameters such as SMD and SATI.

The patterns of adipose tissue accumulation differ markedly according to sex; adipose tissues tend to accumulate in visceral depots in males, while they tend to accumulate in subcutaneous tissues in females [23]. In the general population, the association between fat mass and all-cause mortality reportedly differs between the sexes [10]. Furthermore, a recent study reported that muscle-related BC parameters were prognostic only in male patients with lung cancer, whereas adiposity-related measures were prognostic only in female patients, mirroring our sex-specific observations [11].

In our study, muscle mass/quality and subcutaneous/visceral fat distribution clearly differed between male and female patients. Notably, the impact of BC measures, especially the oncological significance of BC parameters, differed markedly according to sex. Among the examined BC parameters, only a low SMI was an independent predictor of a poor CSS in men, whereas only a low SATI was an independent predictor of a poor CSS in women. Our observations, together with those of prior studies [10, 11], suggest a fundamental biological basis for the interaction between BC, sex hormones, and tumor biology.

Investigators have demonstrated that patients with low subcutaneous adiposity are at high risk of mortality from various malignancies [12], including GEC [24]. In surgical oncology, muscle mass, subcutaneous adiposity, and visceral adiposity should be considered, especially in female patients [7, 25]. Sex differences are equally intriguing in this regard. Estrogen promotes subcutaneous fat deposition and metabolic resilience in women, whereas testosterone deficiency accelerates sarcopenia in men [26]. A previous study demonstrated sex-specific differences in the association between BC and the risk of death from any cause or cardiovascular disease [27]. These hormonal contexts may explain why a low SATI, but not SMI, is a critical determinant in women, whereas the opposite was found to be true in men.

Clinically, our results highlight the importance of integrating BC analyses into preoperative risk stratification and treatment planning. Detailed BC profiling allows clinicians to identify high-risk subgroups that may benefit from targeted interventions, such as resistance training, nutritional supplementation, and/or anti-inflammatory strategies [28].

Although the association between BC and survival varied across age and sex subgroups, formal interaction analyses revealed no statistically significant interactions between BC and either age or sex, suggesting that the prognostic impact of BC was generally consistent across these subgroups. Therefore, the potential age- and sex-specific prognostic effects of BC parameters warrant further investigation in future studies.

Our study has several limitations. The cohort primarily comprised Japanese patients with a relatively low fat mass compared to Western populations, raising questions about the generalizability of our SATI and VATI cut-off values across ethnicities [12, 29]. Thresholds for body composition (BC) parameters vary considerably across studies, which may hinder the accurate estimation of the prevalence of a poor BC status. Therefore, the cutoff values identified in the present study warrant external validation before broader clinical application. Additionally, the inclusion of both EC and GC patients, heterogeneous neoadjuvant regimens, and variations in surgical approach may have introduced confounding factors, although the survival impacts of BC parameters did not differ markedly between EC and GC patients, as shown in Supplementary Fig. 1, Supplementary Tables 4, and Supplementary Table 5. The retrospective, single-center design also limits its external validity. Large, prospective, multicenter studies across diverse populations are warranted to confirm our findings, refine cutoff thresholds, and explore whether sex- and age-specific BC criteria can be applied to the universal prediction of the survival outcomes.

In conclusion, we demonstrated that measures such as skeletal muscle mass, fat distribution, and muscle quality may have distinct prognostic implications according to age and sex. These findings highlight the need to adopt tailored perioperative management approaches that integrate sex- and age-specific BC parameters to improve survival and optimize long-term oncological care. However, the possible age- and sex-specific prognostic effects of BC parameters warrant further investigation in future studies.

## Supplementary Information

Below is the link to the electronic supplementary material.

Supplementary Material 1

## Data Availability

The datasets used and/or analyzed in the current study are available from the corresponding author upon reasonable request.

## Abbreviations

GEC: Gastroesophageal Cancer
BC: Composition
CT: Computed Tomography
GC: Gastric Cancer
EC: Esophageal Cancer
SMI: Skeletal Muscle Index
SMD: Skeletal Muscle Radiodensity
VATI: Visceral Adipose Tissue Index
SATI: Subcutaneous Adipose Tissue Index
HU: Hounsfield Unit
ROC: Receiver Operating Characteristic
OS: Overall Survival
CSS: Cancer-Specific Survival
HR: Hazard Ratio
CI: Confidence Interval
